# Skin-resident CD4^+^ T cells protect against *Leishmania major* by recruiting and activating inflammatory monocytes

**DOI:** 10.1371/journal.ppat.1006349

**Published:** 2017-04-18

**Authors:** Nelson D. Glennie, Susan W. Volk, Phillip Scott

**Affiliations:** 1 Department of Pathobiology, School of Veterinary Medicine, University of Pennsylvania, Philadelphia, Pennsylvania, United States of America; 2 Department of Clinical Studies-Philadelphia, School of Veterinary Medicine, University of Pennsylvania, Philadelphia, Pennsylvania, United States of America; Imperial College London, UNITED KINGDOM

## Abstract

Tissue-resident memory T cells are required for establishing protective immunity against a variety of different pathogens, although the mechanisms mediating protection by CD4^+^ resident memory T cells are still being defined. In this study we addressed this issue with a population of protective skin-resident, IFNγ-producing CD4^+^ memory T cells generated following *Leishmania major* infection. We previously found that resident memory T cells recruit circulating effector T cells to enhance immunity. Here we show that resident memory CD4^+^ T cells mediate the delayed-hypersensitivity response observed in immune mice and provide protection without circulating T cells. This protection occurs rapidly after challenge, and requires the recruitment and activation of inflammatory monocytes, which limit parasites by production of both reactive oxygen species and nitric oxide. Overall, these data highlight a novel role for tissue-resident memory cells in recruiting and activating inflammatory monocytes, and underscore the central role that skin-resident T cells play in immunity to cutaneous leishmaniasis.

## Author summary

Cutaneous leishmaniasis is a neglected tropical disease, causing significant worldwide morbidity. There is no vaccine for this infection, in part because of our limited understanding of the memory T cells that might contribute to immunity. We previously discovered that a population of skin-resident memory CD4^+^ T cells that develop in immune mice enhances the protective immune response against leishmania parasites. Here we show that these skin-resident T cells mediate protection within the first three days of infection. This protection was dependent upon the recruitment of inflammatory monocytes to the challenge site, which reduced the parasite burden in a nitric oxide and reactive oxygen species dependent manner. A series of experiments including blockade of cell recruitment from the blood to the lesions, skin grafts, and parabiosis demonstrated that circulating effector T cells do not contribute to this early protection. Together, these results emphasize that skin-resident CD4^+^ T cells play a primary role in controlling parasites immediately after challenge, which not only indicates the importance of generating these cells in a vaccine, but also expands our understanding of the functions of skin-resident CD4^+^ T cells.

## Introduction

Tissue-resident memory T cells (T_RM_) are critical mediators of immunity against a number of different infections in a variety of different tissues [1–11]. Because they are typically located at barrier surfaces and therefore occupy the initial sites of infection, T_RM_ cells are poised to provide rapid protection. CD8^+^ T_RM_ cells are the best defined tissue-resident T cells, and mediate protection through direct cytotoxicity [12–14], production of cytokines [1, 15], maturation of local innate cells [6], triggering of tissue-wide antiviral signaling [16], and/or the recruitment of additional lymphocytes to the site of infection [15]. CD4^+^ T_RM_ cells remain relatively uncharacterized, although they have been described in the lung, vaginal mucosa, and skin [3–5, 17]. We recently demonstrated that skin-resident CD4^+^ T cells play a critical role in immunity to cutaneous leishmaniasis [18], however the various mechanisms by which CD4^+^ T_RM_ cells mediate protection in the skin remain ill-defined.

Human cutaneous leishmaniasis encompasses a spectrum of diseases caused by the intracellular protozoan parasites. Murine models that mimic aspects of the human disease have proven invaluable for understanding the mechanisms mediating susceptibility and resistance [19]. For example, similar to some forms of human cutaneous leishmaniasis, C57BL/6 mice infected with *Leishmania major* develop lesions that heal over several weeks, and once resolved the mice exhibit immunity to reinfection [19]. Studies in this model have shown that in a primary leishmania infection, innate cells including neutrophils, monocytes, and dendritic cells are rapidly recruited to the site of challenge [20–23]. These cells have the potential to restrict parasite infection [21, 24–26], but they can also be co-opted by the parasites to evade immune detection or suppress the immune response [20, 27, 28]. Conversely, in a secondary infection, the recruitment of pre-existing circulating effector CD4^+^ Th1 cells leads to the rapid control of the parasites [29, 30], and CD4^+^ T_RM_ cells contribute by promoting the recruitment of these effector T cells to the site of infection [18]. However, given their location at the site of a challenge infection and their rapid production of IFNγ, it might be expected that CD4^+^ T_RM_ cells may also provide some level of rapid protection that is independent of additional T cell recruitment from the blood.

Here we show that CD4^+^ T_RM_ cells mediate control of the parasite burden within the first three days of infection, which correlates with a strong delayed-type hypersensitivity (DTH) response, the hallmark of immunity in murine and human leishmaniasis. While IFNγ produced by T_RM_ cells might be expected to activate resident macrophages in the skin and limit the parasite burden, surprisingly we found that protection by CD4^+^ T_RM_ cells required the recruitment of inflammatory monocytes that subsequently controlled the parasites by the induction of both reactive oxygen species (ROS) and inducible nitric oxide synthase (iNOS). Importantly, we found that T_RM_ cells provided protection independently of circulating CD4^+^ T cells, emphasizing the importance of generating T_RM_ cells for optimal immunity to leishmaniasis.

## Results

### *L*. *major* immune mice are protected within 72 hours of challenge in a CD4^+^ T_RM_ cell dependent manner

In experimental models of cutaneous leishmaniasis, protection to a challenge infection is often assessed after several weeks, when a large difference in parasite number is evident between naive and immune mice. This approach also allows for the assessment of protection mediated not only by circulating effector T cells, but also by central memory T cells that are delayed in their protective response [30]. However, the identification of T_RM_ cells and their occupation of the skin led us to hypothesize that they might contribute to immune protection very early after challenge. To test this, we challenged naive and leishmania-immune mice in the ear with *L*. *major*, and assessed the immune response during the first 72 hrs of infection. For these studies, immune mice were infected with *L*. *major* in the contralateral ear at least 12 weeks earlier, and had resolved their primary lesion.

**Fig 1:**
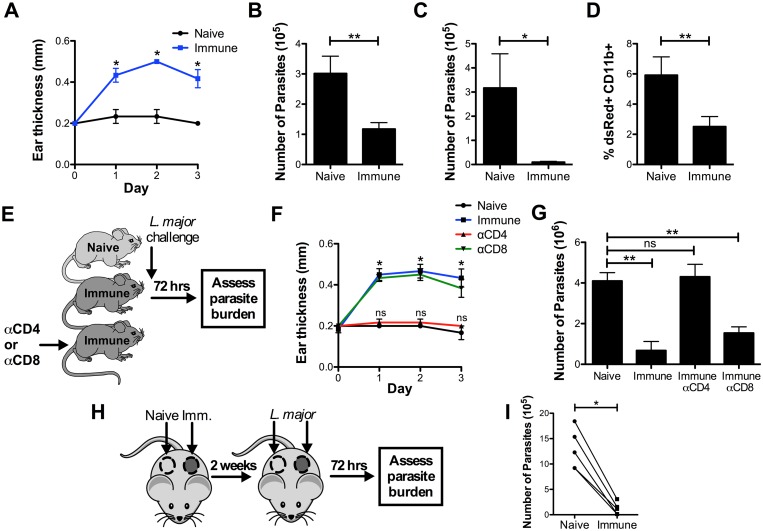
*L*. *major* immune mice are protected within 72 hrs of challenge in a CD4^+^ T_RM_ dependent manner. A) Naive and immune mice were challenged intradermally in the ear with 2x10^6^ dsRed *L*. *major* and DTH response was monitored over the course of 3 days. (B-D) Representative parasite burden in naive and immune mice 72 hrs after challenge as measured by limiting dilution (B), quantitative PCR (C), or flow cytometry (D). Representative data shown are from one experiment representative of eight (n = 3–4 mice per group). E) Naive, immune, and immune mice depleted of CD4^+^ or CD8^+^ T cells were challenged intradermally in the ear with 2x10^6^ dsRed *L*. *major*. F) DTH response was monitored over the course of 3 days. G) Parasite burden was determined at 72 hrs. Data shown are from one experiment representative of two (n = 3 mice per group). H) Naive and immune flank skin were grafted side-by-side onto naive recipients, then each graft was challenged intradermally with 2x10^6^ dsRed *L*. *major*. I) Parasite burden was determined at 72 hrs. Data shown are from one experiment representative of four (n = 5 mice per group). P < 0.05 = *; P < 0.01 = **; P < 0.001 = ***.

One of the hallmarks of immunity to leishmaniasis is the presence of a DTH response, and a positive reaction indicates that an individual has generated a type 1 immune response. As expected, immune mice developed a DTH response, represented by an increase in ear swelling within 24–72 hrs after challenge, while naive mice did not (Fig 1A). In order to evaluate whether the presence of this DTH reaction was associated with control of the challenge inoculum, we assessed the parasite burden by performing three different assays: limiting dilution, qPCR for parasite ribosomal RNA, and analysis of the frequency of cells infected with dsRed expressing parasites by flow cytometry. We found that the number of parasites was consistently decreased 2–4 fold in immune mice at 72 hrs, as measured by limiting dilution and qPCR (Fig 1B and 1C), and that the frequency of infected cells was significantly decreased by flow cytometry (Fig 1D). These results demonstrate that as early as 72 hrs after challenge, mice that have resolved a previous *L*. *major* infection can mount an immune response that is effective at controlling the parasites.

To determine if the DTH in leishmaniasis was dependent on either CD4^+^ or CD8^+^ T cells, we individually depleted each subset in immune mice before challenge with *L*. *major*, and then monitored the DTH response and parasite burden over 72 hrs (Fig 1E). αCD4 treatment, which depletes both circulating and tissue-resident CD4^+^ cells in our hands (S1 Fig), completely ablated the DTH response (Fig 1F), while effective CD8 depletion (S1 Fig) did not, suggesting that CD4^+^ cells are the critical drivers of this early inflammation. Importantly, CD4^+^ cells, but not CD8^+^ T cells, were also required for the decrease in parasites at 72 hrs (Fig 1G).

We next wanted to test if T_RM_ cells were mediating the early control of the parasites. To do so, we grafted naive and immune skin side-by-side onto the flanks of naive recipient mice, challenged each graft, and measured the parasite burden three days later (Fig 1H). As the graft recipients contain only naïve T cells, this approach enabled us to specifically assess the protection mediated by T_RM_ cells, which we have previously shown to remain in the grafted tissue [18]. In all cases, the immune grafts had significantly fewer parasites than their naive counterparts at 72 hrs (Fig 1I). Taken together, these results indicate that CD4^+^ T_RM_ cells mediate parasite protection in immune skin at 72 hrs in a process that is independent of circulating CD4^+^ and CD8^+^ T cells.

### Rapid protection in immune mice is associated with recruitment of inflammatory monocytes

**Fig 2:**
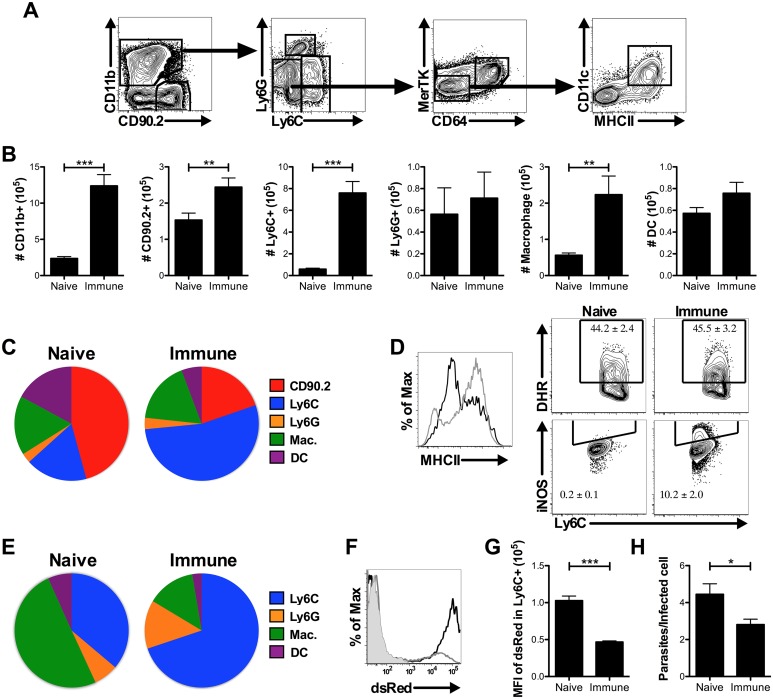
Rapid protection in *L*. *major* immune mice is associated with recruitment of inflammatory monocytes. A) Gating strategy for distinguishing myeloid cell populations. B) Numbers of T cells and myeloid cell populations in ear skin 72 hrs after challenge. C) Proportions of total CD45^+^ population in naive and immune ears 72 hrs after challenge. Data shown are combined from six experiments (n = 18 mice per group). D) Representative histogram or contour plots showing frequency of MHCII^+^, DHR^+^, and iNOS^+^ monocytes in naive (black line) and immune (gray line) mice 72 hrs after challenge. Data shown are from one experiment of two (n = 3 mice per group). E) The proportion of infected CD11b^+^ dsRed^+^ cells comprised of each cell type 72 hrs after challenge. Data shown are combined from six experiments (n = 18 mice per group) F) Representative plot of dsRed expression in CD11b^+^ cells from uninfected (gray fill), naive (black line), or immune (gray line) mice. G) Quantification of dsRed MFI in Ly6C^+^ cells from naive or immune skin 72 hrs after challenge. Data shown are combined from two experiments (n = 8 mice per group). H) Number of parasites per infected cell counted from 50 cells per cytospin slide. Data shown are combined from two experiments (n = 5–6 mice per group). P < 0.05 = *; P < 0.01 = **; P < 0.001 = ***.

To gain further insight into how this rapid protection is mediated, we analyzed the cells recruited to the skin of naive and immune mice 72 hrs after challenge. We compared the numbers of CD90.2^+^ T cells, Ly6G^+^ neutrophils, Ly6C^+^ inflammatory monocytes, MerTK^+^ CD64^+^ macrophages, and CD11c^+^ MHCII^+^ dendritic cells in naive and immune skin 72 hrs after infection (Fig 2A). As expected, we observed increased T cell recruitment to immune skin consistent with previous results [18, 29]. However, a majority of the recruited cells were myeloid lineage cells, specifically inflammatory monocytes (Fig 2B and 2C). We analyzed the activation status of these monocytes and found that they expressed high levels of MHCII, ROS, and iNOS. Further, both MHCII and iNOS expression were significantly increased in the monocytes recruited to immune skin compared with those recruited to naive skin (Fig 2D). Finally, using fluorescent parasites, we found that greater than 70% of the infected cells in the skin of immune mice were inflammatory monocytes (Fig 2E). Notably, these infected cells contained fewer parasites per cell when compared with monocytes in naive skin, as demonstrated by the lower MFI of dsRed (Fig 2F and 2G), and when counted in cytospins (Fig 2H). These data show that monocytes are highly recruited to immune skin where they are more likely to be infected than other cell types, have a more activated phenotype, and contain fewer parasites per infected cell. These results suggest that inflammatory monocytes recruited by T_RM_ cells might be better able to kill parasites, and therefore we next investigated whether they were required for parasite control and if so how they mediated protection.

### Early protection is dependent on inflammatory monocytes

**Fig 3:**
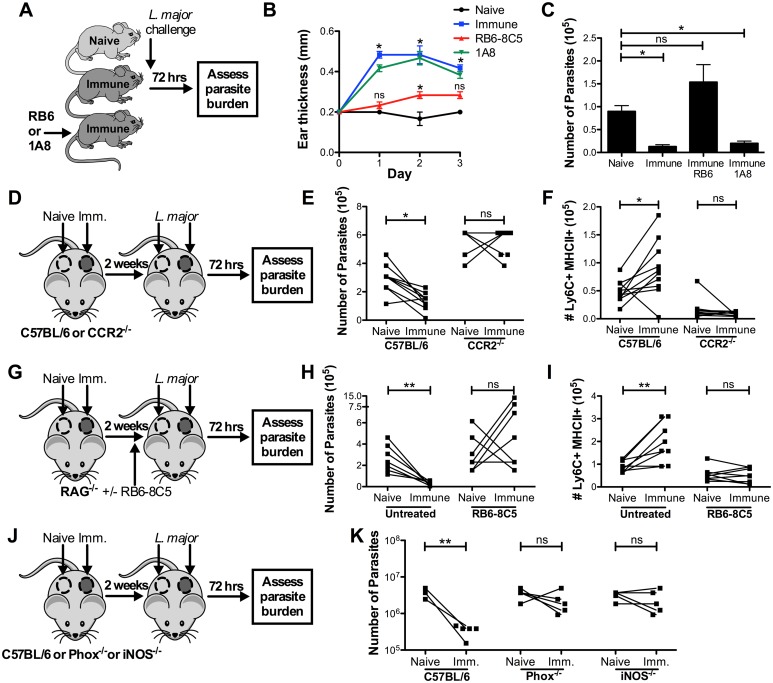
Early protection is dependent on inflammatory monocytes. A) Naive, immune, and immune mice depleted with αGR1 clone RB6-8C5 (neutrophils and monocytes) or 1A8 (neutrophils only) were challenged intradermally in the ear with 2x10^6^ dsRed *L*. *major*. B) DTH response was monitored over the course of 3 days. C) Parasite burden at 72 hrs was determined by limiting dilution. Data shown are from one experiment representative of two (n = 3 mice per group). D) Naive and immune flank skin were grafted side-by-side onto WT or CCR2^-/-^ naive recipients, then each graft was challenged intradermally with 2x10^6^ dsRed *L*. *major*. E, F) Parasite burden was determined in each graft by limiting dilution (E), and the number of activated Ly6C^+^MHCII^+^ monocytes was determined at 72 hrs (F). Data shown are combined from two experiments (n = 9–10 mice per group). G) Naïve and immune flank skin were grafted side-by-side onto RAG^-/-^ recipients. Half the mice were treated with 500μg αGR1 clone RB6-8C5 one day prior to challenge, then each graft was challenged with 2x10^6^ dsRed *L*. *major*. H) Parasite burden was determined in each graft by limiting dilution at 72 hrs. I) The number of Ly6C^+^ MHCII^+^ cells in each graft was quantified. Data shown is combined from two experiments (n = 4 mice per group). J) Naive and immune flank skin were grafted side-by-side onto WT, PHOX^-/-^, or iNOS^-/-^ naive recipients, then each graft was challenged with 2x10^6^ dsRed *L*. *major*. K) Parasite burden was determined in each graft by limiting dilution at 72 hrs. Data shown are from one experiment representative of two (n = 5 mice per group). P < 0.05 = *; P < 0.01 = **; P < 0.001 = ***.

To assess the role of inflammatory monocytes in the early protection of immune mice, we used a pair of depleting antibodies that target either Ly6G^+^ and Ly6C^+^ cells (and thus deplete both neutrophils and monocytes) or Ly6G^+^ cells alone (and thus deplete only neutrophils) (Fig 3A, S2 Fig). Depletion of neutrophils alone had no effect on the DTH response in immune mice, but depletion of both neutrophils and monocytes dramatically reduced the early inflammatory response (Fig 3B). Importantly, the protection observed in immune mice was also completely ablated by the depletion of monocytes and neutrophils, while depleting neutrophils alone did not have a significant effect (Fig 3C). Because activated CD4^+^ cells can also express Ly6C, we confirmed that RB6-8C5 treatment did not reduce the frequency of Ly6C^+^ CD4^+^ T cells in the spleen or ear after challenge (S3 Fig), or the frequency of leishmania-specific IFNγ^+^ skin-resident T cells (S4 Fig), which are intermediate for Ly6C expression (S5 Fig). Taken together, these data suggest that inflammatory monocytes are the critical mediators of early protection.

To specifically address whether inflammatory monocyte recruitment is critical to early protection against *L*. *major*, we assessed the response to challenge in CCR2^-/-^ mice, which contain monocytes that lack the ability to respond to CCL2 and CCL7 chemokine signaling and therefore cannot be efficiently recruited to sites of inflammation [31, 32]. To do so, we grafted naive and immune skin from WT mice onto the flanks of naive WT or CCR2^-/-^ recipients, challenged with *L*. *major*, and measured the parasite burden 72 hrs later (Fig 3D). As previously observed, immune skin had significantly fewer parasites compared to naive skin in WT recipient mice (Fig 3E). In contrast, the reduction of parasites in immune skin was lost in CCR2^-/-^ recipient mice (Fig 3E), and correlated with a loss of activated monocytes in the skin (Fig 3F). Together, these results demonstrate that it is recruited CCR2^+^ monocytes, rather than resident myeloid cells, that are required for protection.

To further confirm that inflammatory monocytes were necessary for early protection, and that this protection could be conferred in the absence of circulating T cells, we grafted WT naïve and immune skin onto the flanks of RAG^-/-^ recipient mice that lack T and B lymphocytes. Additionally, we treated some of the mice with αGR1 clone RB6-8C5 to deplete inflammatory monocytes and neutrophils as described above. We challenged each graft with *L*. *major* and measured the parasite burden 72 hrs later (Fig 3G). As expected, immune grafts on RAG^-/-^ mice contained significantly fewer parasites, demonstrating that the protection observed at 72 hrs was independent of circulating lymphocytes (Fig 3H). Protection in immune skin was lost in mice treated with RB6-8C5 (Fig 3H). When we quantified the number of Ly6C^+^MHCII^+^ cells in each graft, we found a strong correlation with the level of protection (Fig 3I). Taken together, these data further implicate inflammatory monocytes as the critical cell type required for early protection.

To gain further insight into the mechanism by which the inflammatory monocytes might control the parasites, we performed skin graft experiments in which we grafted naive and immune skin onto the flank of 1) WT naive mice, 2) Phox^-/-^ mice in which monocytes lack the ability to produce ROS, or 3) iNOS^-/-^ mice that have deficient NO production (Fig 3J). Grafts were then challenged with *L*. *major*, and the parasite burden measured at 72 hrs. We found that the protection associated with immune skin was lost in both the Phox^-/-^ and iNOS^-/-^ mice (Fig 3K), suggesting that both ROS and NO from inflammatory monocytes are required for this early protection.

### Early protection is not enhanced by circulating memory T cells

**Fig 4:**
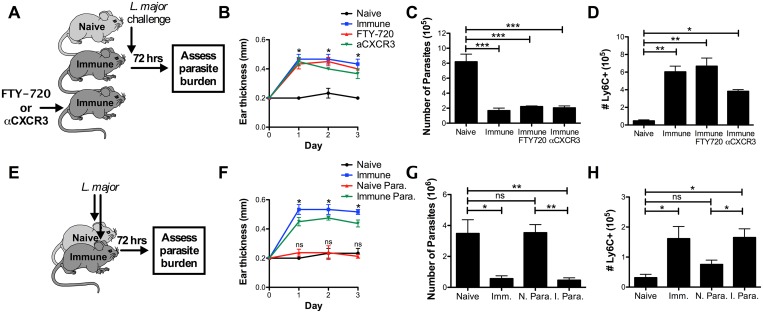
Early protection is not enhanced by circulating memory T cells. A) Naive, immune, and immune mice treated with FTY-720 or αCXCR3 were challenged intradermally in the ear with 2x10^6^ dsRed *L*. *major*. B) DTH response was monitored over the course of 3 days. C, D) Parasite burden (C) and the number of Ly6C^+^ monocytes in the infection site (D) was determined at 72 hrs. Data shown are from one experiment representative of two (n = 3 mice per group). E) Naive, immune, naive parabiotic, and immune parabiotic mice were challenged intradermally in the ear with 2x10^6^ dsRed *L*. *major*. F) DTH response was monitored over the course of 3 days. G, H) Parasite burden (G) and the number of Ly6C^+^ monocytes in the infection site (H) was determined at 72 hrs. Data shown are from one experiment representative of two (n = 3 mice per group). P < 0.05 = *; P < 0.01 = **; P < 0.001 = ***.

Although T_RM_ cell-mediated recruitment of inflammatory monocytes was sufficient to reduce the parasite burden at 72 hrs, we predicted that the presence of circulating leishmania-specific T cells might further enhance immunity, as they are also recruited early after challenge [18, 29]. To examine the contribution of circulating T cells, we pretreated immune mice with either FTY-720, which prevents egress of T cells from tissues, or αCXCR3, which we previously demonstrated blocks the ability of T_RM_ cells to recruit effector T cells from circulation [18] (Fig 4A). Despite the expected decrease in the number T cells recruited to the challenge site (S6 Fig), neither treatment affected the DTH response (Fig 4B), the decrease in parasite burden (Fig 4C), or the recruitment of monocytes (Fig 4D). Unexpectedly, this result demonstrates that DTH and early parasite control are not enhanced by T cells from circulation, and implies that CD4^+^ T_RM_ cells are solely responsible for mediating these responses.

To test if circulating T cells would provide protection in the absence of T_RM_ cells, we utilized a parabiotic model in which the circulations of naive and immune mice were surgically joined, allowing circulating T cells to equilibrate between the two animals, while T_RM_ cells remained exclusively in the immune partner (Fig 4E, S7 Fig). Each parabiont was then challenged with *L*. *major* in the ear, and DTH and parasite number were measured 72 hrs later. As expected, immune parabionts had the same DTH response, monocyte recruitment, and parasite numbers as control immune mice (Fig 4F–4H). In contrast, naive parabionts, despite having a full complement of circulating memory T cells, did not exhibit a DTH response, had defective monocyte recruitment, and lost the early protection observed in immune mice (Fig 4F–4H). These results show that circulating leishmania-specific T cells by themselves are unable to provide any protection at this early time point, further demonstrating that T_RM_ cells are the critical subset for this rapid protection.

### Circulating memory T cells are not required to control low dose *L*. *major* infection

In contrast to the parasite control observed at 72 hrs in immune mice, we previously found that when protection was assessed two weeks after challenge with 2 x 10^6^ parasites, optimal parasite control depended upon both CD4^+^ T_RM_ cells and circulating effector T cells [18]. These results, in combination with our current findings, suggest that while T_RM_ cells may initially reduce the parasite number, the long-term consequences are limited in the absence of additional circulating T cells. However, since parasite dose can significantly influence what is required for protection, and the number of infective parasites transmitted by the sand fly is thought to be much lower than 2 x 10^6^ parasites [33], we next tested whether T_RM_ cells might provide protection greater than 72 hrs after challenge if fewer parasites were present in the challenge inoculum.

**Fig 5:**
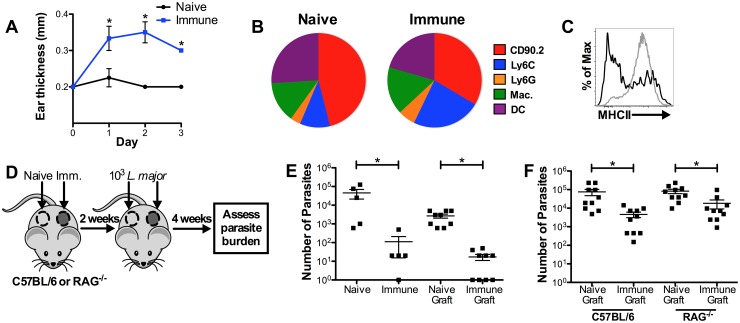
Circulating memory T cells are not required to control low dose *L*. *major* infection. A) Naive and immune mice were challenged in the ear with 10^3^ parasites and DTH was monitored over 72 hrs. B) Proportions of total CD45^+^ population in naive and immune ears 72 hrs after low dose challenge. C) Representative histogram showing frequency of MHCII cells in naive (black line) and immune (gray line) mice 72 hrs after low dose challenge. Data shown are from one experiment of two (n = 3 mice per group). D) Naive and immune flank skin were grafted side-by-side onto naive WT or RAG^-/-^ mice, then each graft was challenged with 10^3^ dsRed *L*. *major*. E) Parasite burden at 4 weeks was compared between intact naive and immune mice as well as naive and immune grafts on WT recipients. Data shown are from one experiment representative of two (n = 5 or 9 mice per group). F) Parasite burden at 4 weeks was compared between naive and immune grafts on WT or RAG^-/-^ recipients. Data shown are combined from two experiments (n = 5 mice per group). P < 0.05 = *; P < 0.01 = **; P < 0.001 = ***.

First, we challenged naive and immune mice with 10^3^ parasites in the ear, measured the DTH, and assessed the composition of cells recruited to the challenge site at 72 hrs. Similar to our results with high dose challenge, immune mice had an increased DTH response (Fig 5A), there was a large population of inflammatory monocytes infiltrating the lesions (Fig 5B), and the monocytes had a more activated phenotype (Fig 5C), though the magnitude of the overall response was lower.

To test if T_RM_ cells could provide protection more than 72 hrs after challenge without circulating T cells, we challenged intact naive mice, immune mice, naive grafts, and immune grafts with 10^3^ *L*. *major*, and measured parasite burdens 4 weeks later (Fig 5D). As expected, intact immune mice were better protected than naive mice against low dose *L*. *major* challenge (Fig 5E). However, immune skin grafts also showed significantly better protection than their naive counterparts, despite the absence of circulating leishmania-specific effector T cells (Fig 5E). These results demonstrate that T_RM_ cells do not require previously activated circulating T cells to provide protection as long as 4 weeks after low dose challenge. However, naive leishmania-specific T cells would be expanded during the 4 weeks of infection, and could contribute to the protection we measured. Therefore, to test if T_RM_ cells could provide protection without any circulating lymphocytes, we grafted naive and immune skin onto WT and RAG^-/-^ recipient mice, challenged with 10^3^ parasites, and measured the parasite burden 4 weeks later (Fig 5D). Surprisingly, we found that the immune grafts showed significantly better control of the parasites in both WT and RAG^-/-^ mice (Fig 5F). While circulating effector T cells may have potential to contribute to long-term immunity, these results indicate that T_RM_ cells and innate cells alone are sufficient to provide a significant level of protection.

## Discussion

We recently reported that T_RM_ cells provide optimal immunity against *L*. *major* infection by recruiting circulating leishmania-specific effector T cells to the site of infection [18]. We now identify an additional, novel function for leishmania-specific T_RM_ cells: to rapidly recruit and activate inflammatory monocytes at the site of infection, resulting in a significant reduction in the initial parasite burden. Further, we show that when the challenge inoculum is at a physiologically relevant dose, CD4^+^ T_RM_ and inflammatory monocytes exhibit significant control of the parasites, even when circulating leishmania-specific effector T cells are not present. Together, these results demonstrate that in addition to facilitating the recruitment of circulating effector T cells, CD4^+^ T_RM_ cells play a primary role in controlling parasites immediately after challenge, which not only indicates the importance of generating CD4^+^ T_RM_ cells in a vaccine, but also expands our understanding of the functions of CD4^+^ T_RM_ cells.

Our experiments have identified CD4^+^ T_RM_ cells as the critical cell subset required for both the DTH response and the immediate control of leishmania infection. The response is antigen specific as it is not induced by PBS injection, and is likely initiated via local antigen presentation [34]. The identification of T_RM_ cells as required for DTH responses was unexpected, since the prevailing view was that circulating effector T cells mediated DTH. However, our results are similar to those that have been described in studies of contact-hypersensitivity, where T_RM_ cells mediated the inflammatory response independent of circulating T cells [35]. Since DTH responses can be elicited at sites distal to the initial site of infection, these results confirm that CD4^+^ T_RM_ are distributed and can function throughout the skin. Thus, our results extend those of others which have focused on the functions of CD4^+^ T_RM_ cells at the site of infection [3, 17]. Surprisingly, neither CD8^+^ T cells nor circulating effector T cells are required for the DTH response and the early control of the parasites, implicating CD4^+^ T_RM_ as the mediators of the initial inflammatory responses.

We found that inflammatory monocytes are rapidly recruited to the lesion site by T_RM_ cells, and are responsible for the observed protection. Inflammatory monocytes are important mediators of protection against many viral [36, 37] bacterial [38–40], fungal [41, 42], and parasitic infections [24, 43, 44], and thus this mechanism of protection has the potential to influence a number of different immune responses. Inflammatory monocytes are known to be activated by memory T cells [45], and restrict leishmania infection in certain contexts [21, 24, 44], but their role in secondary leishmania infection and interaction with T_RM_ cells has not previously been appreciated. On the other hand, neutrophils did not appear to be required for early protection or the recruitment of inflammatory monocytes. This is in contrast to a primary infection where neutrophils may contribute to the recruitment of dendritic cells [46]. This difference is most likely due to the presence of T_RM_ cells, which are sufficient to mediate phagocyte recruitment in secondary challenge. Indeed, CCL2 and CCL7 transcripts are both increased 12 hrs after challenge in immune mice [18], and CCR2 signaling is required for the recruitment of inflammatory monocytes and subsequent protection.

ROS and NO from myeloid lineage cells have both been shown to have roles in controlling leishmania infections, though results vary based on the site of infection, the species of parasite involved, and whether the studies are done in mice or humans [21, 47–51]. Nonetheless, it is clear in our model that both ROS and NO are required for full protection early after challenge of immune mice, and neither is sufficient alone. Thus, while most studies in mice have emphasized the central role of NO, it has become clear that ROS can contribute to protection not only in humans but also in the mouse. For example, following *L*. *major* infection Phox^-/-^ mice can develop chronic lesions long after presumed cure [47]. Understanding why ROS is required under certain conditions for control of leishmanial infections in mice is still not well understood, although we would speculate that at the early time point the levels of NO induced may be insufficient for parasite control, and ROS are required to boost the killing by the inflammatory monocytes.

Currently there is no human vaccine for leishmaniasis, which has been partially attributed to the inability to maintain sufficient circulating effector T cells following immunization [52–54]. Thus, it is the presence of low numbers of parasites in immune mice that are believed to maintain maximal levels of responsive effector cells [29, 30, 52, 54]. However, our studies indicate that T_RM_ cells can mediate protection alone, suggesting that these T cells should be targeted for a vaccine. Importantly, we found that they can survive in the absence of persistent parasites [18], similar to central memory T cells [30], and thus if generated in a vaccine may be maintained long-term. Thus, defining the requirements for the generation and maintenance of T_RM_ cells, as well as developing vaccination strategies that induce T_RM_, are the next important steps in developing a vaccine for leishmaniasis.

As our understanding of tissue resident T cells grows, more functions have been attributed to T_RM_ cells. CD8^+^ T_RM_ can be directly cytotoxic [12, 13], and IFNγ from T_RM_ cells has been shown to drive recruitment of circulating T cells [15, 18]. Transcriptional analyses have identified a core set of changes induced by T_RM_ activation that induce a tissue state of pathogen alert, capable of protecting against viral challenge non-specifically, [6, 16]. However, this is the first demonstration to our knowledge of T_RM_ cells orchestrating the innate response and classic DTH responses by recruiting inflammatory monocytes to the site of infection. This protective mechanism has the potential to be relevant for a number of different intracellular infections, as DTH responses are the hallmark of immunity against many infections and inflammatory monocytes are potent killers of many pathogens [55]. Although circulating effector T cells are undoubtedly beneficial, in certain contexts the rapid response provided by a combination of CD4^+^T_RM_ cells and inflammatory monocytes that lessen the initial pathogen burden may be critical in limiting the magnitude of the disease.

## Materials and methods

### Ethics statement

This study was conducted according to the principles specified in the Declaration of Helsinki and under local ethical guidelines (University of Pennsylvania Institutional Review Board). This study was carried out in strict accordance with the recommendations in the Guide for the Care and Use of Laboratory Animals of the National Institutes of Health. The protocol was approved by the Institutional Animal Care and Use Committee, University of Pennsylvania Animal Welfare Assurance Number 805186.

### Mice

C57BL/6 mice were purchased from the National Cancer Institute (Fredericksburg, MD). CCR2-/- (B6.129S4-*Ccr2*^*tm1Ifc*^/J), Phox-/- (B6.129S-*Cybb*^*tm1Din*^/J), iNOS-/- (B6.129P2-*Nos2*^*tm1Lau*^/J), and RAG-/- (B6.129S7-*Rag1*^*tm1Mom*^/J) mice were purchased from The Jackson Laboratory. All mice were maintained in a specific pathogen-free environment at the University of Pennsylvania Animal Care Facility.

### Parasites

*L*. *major* (Friedlin) or dsRed^+^ *L*. *major* (Friedlin) parasites were grown in complete Schneider's insect medium (GIBCO) supplemented with 20% heat-inactivated FBS, 2mM glutamine, 100 U/ml penicillin, 100 μg/ml streptomycin, and 50 μg/mL G418 sulfate (Cellgro) (CSM). Metacyclic enriched promastigotes were used for infection [56]. Mice were infected with 2 x 10^6^ *L*. *major* or 10^3^ *L*. *major* intradermally in the ear or flank skin as noted.

### Antibodies and treatments

For flow cytometry analysis αCD45 APC-eF780, αCD45.2 FITC, αCD45.1PE-Cy7, αCD90.2 BV605, αCD11b BV650, αCD4 PE TexasRed, αCD8b PerCp/Cy5.5, αLy6C AF700, αLy6G PacBlue, αMerTK APC, αCD64 PE-Cy7, αCD11c FITC, αMHCII APC, αAF488 iNOS were incubated with single cell suspensions 30 minutes at 4°C and read on LSR Fortessa. For ROS stain, 2ng/mL dihydrorhodamine 123 (DHR, Cayman Chemical) was added directly ex vivo, then incubated 30 minutes at 37°C for 30 minutes. For in vivo blockade/depletion 250 μg of αCD4 (GK1.5), αCD8 (53–6.72), αCXCR3 (CXCR3-173), 500 μg of αGR1 (RB6-8C5), αLy6G (1A8) (BioXcell), or 1 mg/kg FTY-720 (Cayman Chemical) were given i.p. one day before challenge.

### Skin preparation

For ear preparation, dorsal and ventral layers of the ear were separated and incubated in RPMI (Gibco) with 250 μg/mL Liberase TL (Roche) for 90 minutes at 37°C in 5% CO_2_. Skin was then dissociated using a 40 μm cell strainer (BD Pharmingen) and resuspended in complete RPMI media (cRPMI) containing 10% FBS, 100 U/ml penicillin, 100 μg/ml streptomycin, and 55 μM 2-Mercaptoethanol. For flank skin preparation, a section of skin was harvested from the flank following hair removal with an electric trimmer equipped with a two-hole precision blade (Wahl). Skin sections were then minced with a sterile scalpel blade into ~2mm sections, and incubated in RPMI containing 1 mg each of type III and type IV collagenase (Worthington) for 120 minutes with vortexing every 30 minutes. The resulting solution was passed through a 40 μm cell strainer and resuspended in cRPMI. Bone marrow derived dendritic cells for restimulations were generated by culturing C57BL/6 bone marrow in GM-CSF supplemented cRPMI for 7–11 days. BMDCs were then harvested and infected 5–8 hours with stationary phase *L*. *major* at a ratio of 10:1 in the presence of 1 μg/ml CpG and LPS. Infected BMDCs were incubated at a ratio of 1:5 with 10^6^ skin cells in 24 well plates for 12–16 hours. Cells were incubated for the last 4 hours with 5 μg/ml BFA (eBioscience), stained for IFNγ, and analyzed by flow cytometry.

### Parasite quantification

Parasite burden from ear and flank skin was calculated by serial 2-fold dilution in 96-well plates of CSM and incubated at 26°C. The number of viable parasites was calculated from the highest dilution at which parasites were observed 7 days into culture. For qPCR, single cell suspensions from infected tissue were diluted in RLT lysis buffer, then RNA was isolated using the RNeasy Plus kit (Qiagen). RNA was converted to cDNA using the High Capacity RNA to cDNA kit (Applied Biosciences), then the Power SYBR green PCR mater mix (Applied Biosciences) was used to quantify parasite ribosomal ssRNA on the ViiA7 qPCR machine (Applied Biosciences) with primers F: 5'-TACTGGGGCGTCAGAG-3' and R: 5'-GGGTGTCATCGTTTGC-3'. Cytospins were prepared at 1000 RPM (Shandon Cytospin3) and imaged by light microscopy at 40X magnification (Nikon E600).

### Skin grafts

Skin grafts were performed as previously described [18]. Briefly, donor skin was prepared under sterile conditions from naive and immune mouse flank skin by shaving, depilating, cleaning with chlorhexidine (Vetoquinol), then excising the skin using sterile 8mm biopsy punches (Miltex). Grafts were placed onto a fresh graft bed prepared by excising skin using a 6mm biopsy punch. All mice were anesthetized, received analgesics, and were monitored post-operatively as previously described. In challenge experiments, graft skin was injected intradermally with 2 x 10^6^ metacyclic *L*. *major* 14–20 days after grafting.

### Parabiosis

Congenically disparate (CD45.1^+^ naive and CD45.2^+^ immune) mice were cohoused 2 weeks prior to surgery. After induction of anesthesia with isoflorane, each received 0.1mg/kg buprenorphine subcutaneously as preemptive analgesia. The surgical site was shaved and aseptically prepared with chlorhexidine scrub. A longitudinal skin incision was made on the mirroring side in each mouse starting at 0.5 cm above the elbow and ending 0.5 cm below the knee joint. The left elbow and knee of one animal were attached to the right elbow and knee of the other with a 3–0 ethilon suture (Ethicon) around each joint beneath the skin in a manner loose enough to not disrupt circulation to the distal limb. The dorsal and ventral skin edges created by the flank incision from one mouse were sutured to the respective skin edges of the second mouse using a continuous absorbable 5–0 vicryl suture patter (Ethicon). Suture glue (Abbott laboratories) was used to approximate skin edges. 0.5 ml of 0.9% NaCl was administered subcutaneously to each mouse to prevent dehydration in the immediate post-operative recovery period, and mice were monitored twice daily for the first 48 hrs post-operatively, then observed daily for signs of surgical site complications, pain, or discomfort. In challenge experiments, ears were infected intradermally with 2 x 10^6^ metacyclic *L*. *major* 14–20 days after surgery.

### Statistical analysis

Statistical analysis was performed with the Student's t-test (paired or unpaired where applicable), ANOVA, or 2-way ANOVA in Prism software (GraphPad).

## Supporting information

S1 Fig Efficacy of CD4 and CD8 depletions. Frequency or number of CD4^+^ and CD8^+^ cells in the spleen and challenged ear 72 hours after infection of CD4 and CD8 depleted immune mice are shown.
(TIF)

S2 Fig Efficacy of RB6-8C5 and 1A8 depletions. Number of Ly6C^+^ and Ly6G^+^ cells in the challenged ear 72 hours after infection of RB6-8C5 or 1A8 treated immune mice are shown.
(TIF)

S3 Fig RB6-8C5 treatment does not ablate circulating Ly6C^+^ CD4^+^ T cells. Frequency of Ly6C^+^ CD4^+^ T cells in the spleen and challenged ear 72 hours after infection of RB6-8C5 or 1A8 treated immune mice are shown.
(TIF)

S4 Fig RB6-8C5 treatment does not deplete T_RM_ cells. The frequency of T_RM_ cells, as represented by IFNγ^+^ CD4^+^ T cells in the flank skin upon restimulation with *L*. *major* infected BMDCs, is shown for immune mice treated with 500μg RB6-8C5 or 1A8.
(TIF)

S5 Fig Ly6C expression is intermediate on T_RM_ cells. Comparison of Ly6C MFI on naïve or Ly6C^+^ effector cells from the blood and T_RM_ cells from the flank, as represented by cells that produced IFNγ in response to restimulation with *L*. *major* infected BMDCs.
(TIF)

S6 Fig Efficacy of FTY-720 and αCXCR3 blockade. Frequency or number of CD4^+^ and CD8^+^ cells in the blood and challenged ear 72 hours after infection of FTY-720 or αCXCR3 treated immune mice are shown.
(TIF)

S7 Fig Characterization of parabiosis model. (Top left) Proportions of CD4^+^ and CD8^+^ T cells of naïve (white) or immune (black) origin found in naïve parabionts 2.5 weeks after joining. (Top right) Representative plots showing frequency of leishmania-specific, IFNγ^+^ cells in the blood and flank of naive and immune parabionts 2.5 weeks after surgery upon restimulation with *L*. *major* infected BMDCs. (Bottom) Combined data showing frequency of IFNγ^+^ cells in the blood and flank of naive and immune parabionts 2.5 weeks after surgery upon restimulation with *L*. *major* infected BMDCs, as well as frequency of immune origin Ly6C^+^ CD4^+^ T cells in naïve and immune parabionts.
(TIF)
