# Supplementary figures and images for: Skin-resident CD4+ T cells protect against Leishmania major by recruiting and activating inflammatory monocytes
Source: PLoS Pathog. 2017 Apr 18;13(4):e1006349. doi: 10.1371/journal.ppat.1006349 (PMC5409171; doi:10.1371/journal.ppat.1006349)

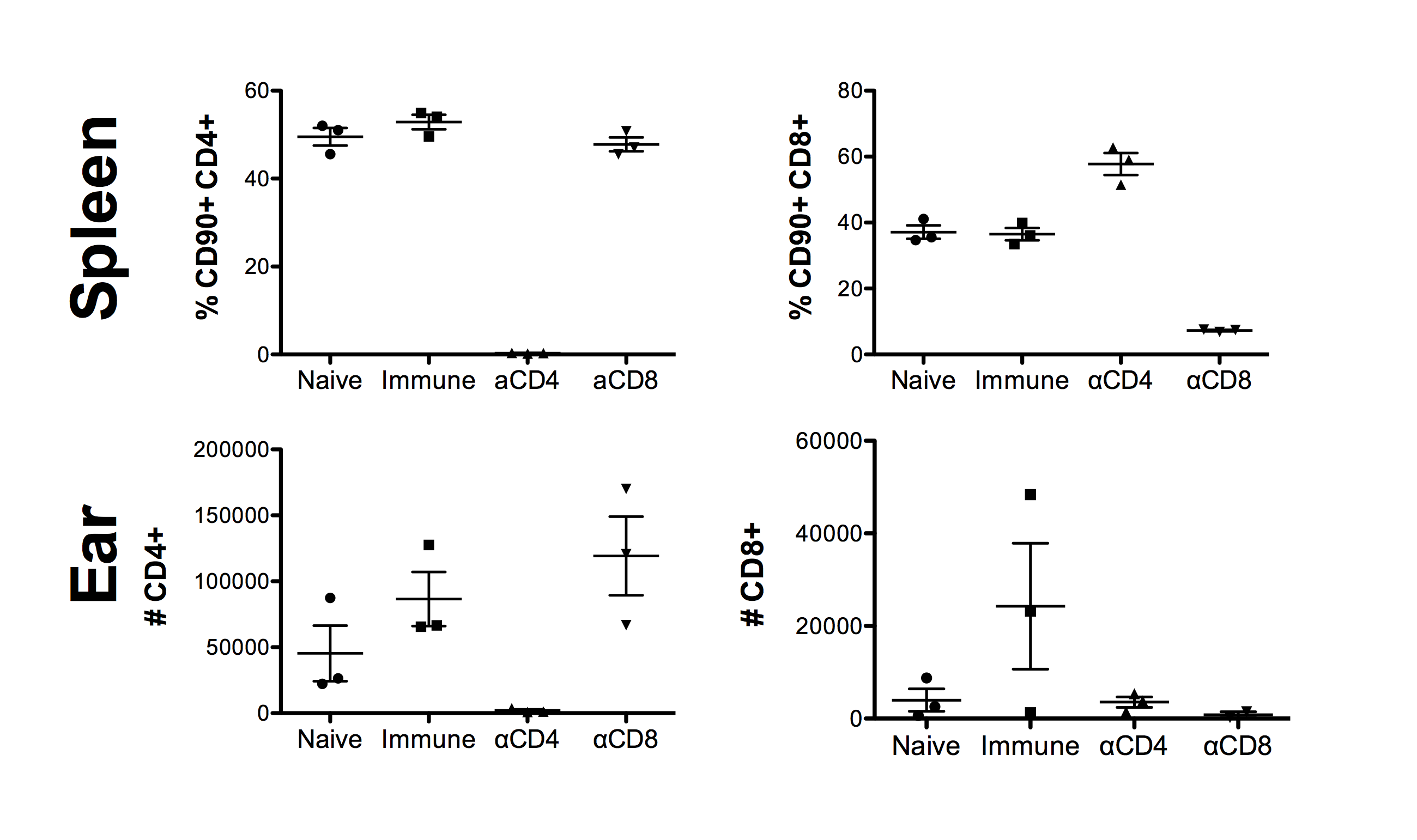

Supplement: S1 Fig — Frequency or number of CD4+ and CD8+ cells in the spleen and challenged ear 72 hours after infection of CD4 and CD8 depleted immune mice are shown. (TIF) [file ppat.1006349.s001.tif]

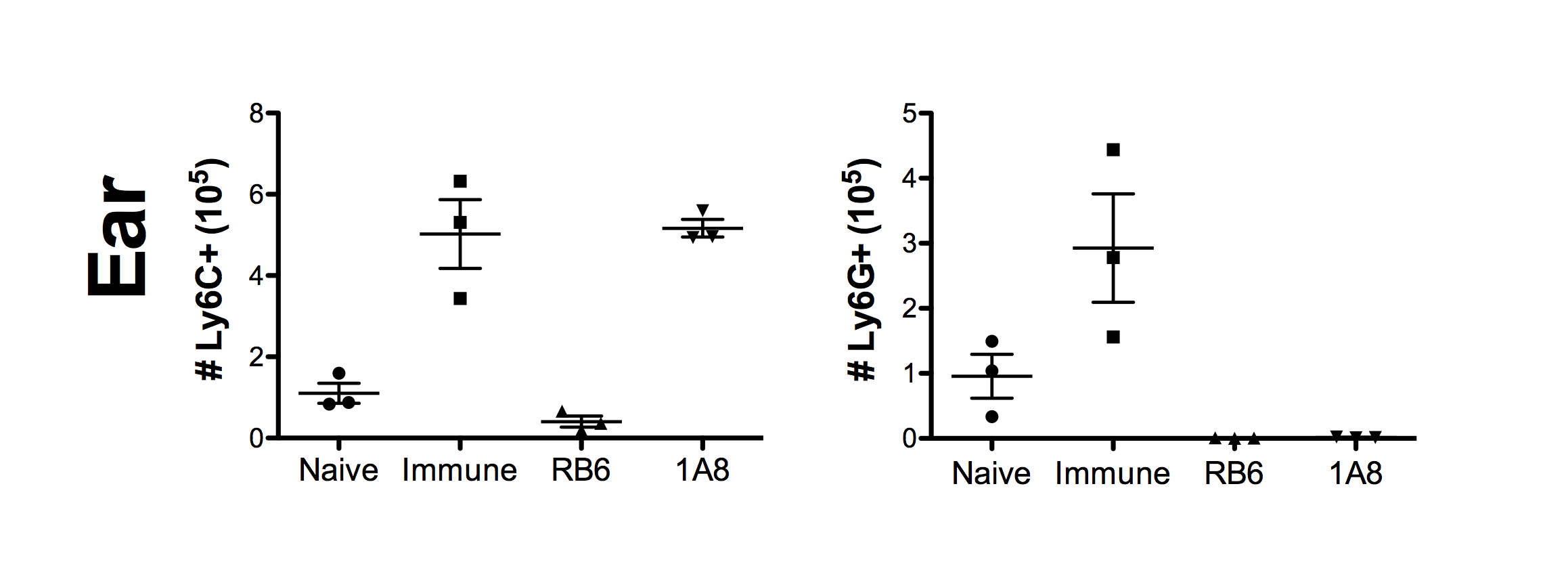

Supplement: S2 Fig — Number of Ly6C+ and Ly6G+ cells in the challenged ear 72 hours after infection of RB6-8C5 or 1A8 treated immune mice are shown. (TIF) [file ppat.1006349.s002.tif]

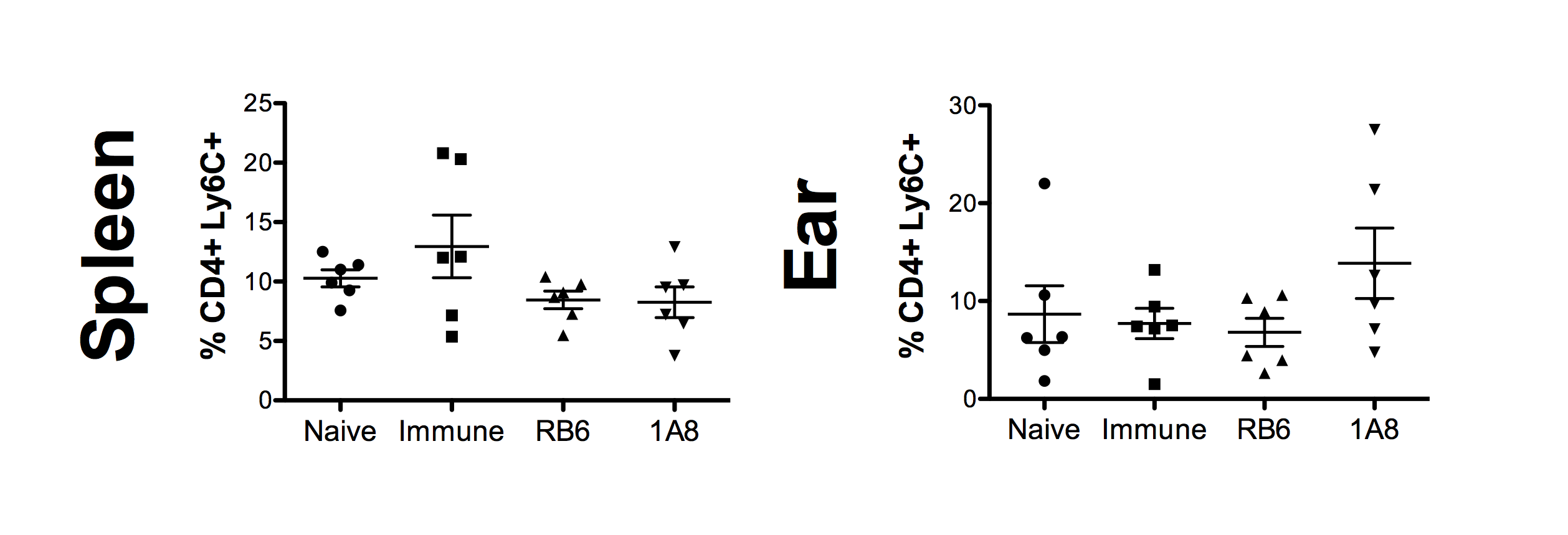

Supplement: S3 Fig — Frequency of Ly6C+ CD4+ T cells in the spleen and challenged ear 72 hours after infection of RB6-8C5 or 1A8 treated immune mice are shown. (TIF) [file ppat.1006349.s003.tif]

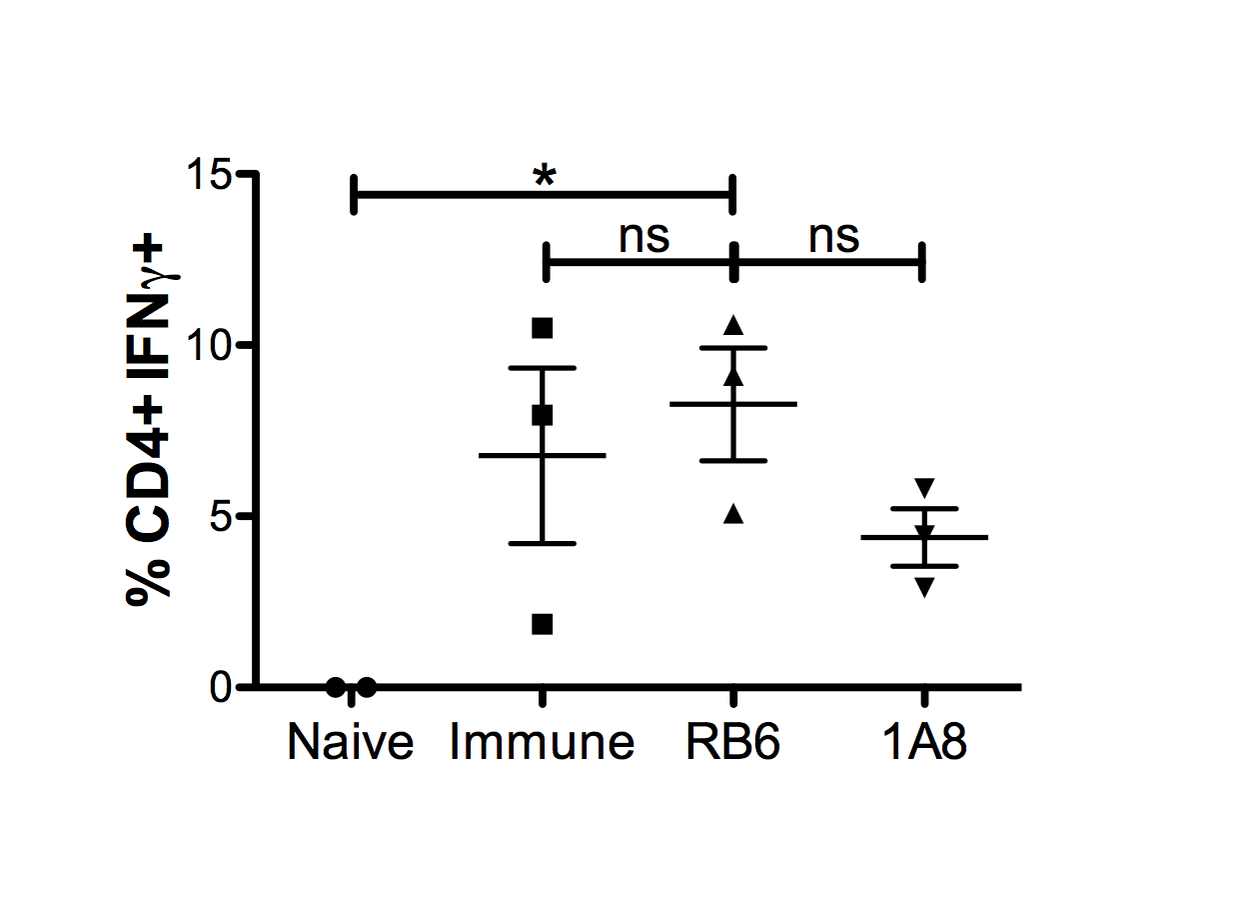

Supplement: S4 Fig — The frequency of TRM cells, as represented by IFNγ+ CD4+ T cells in the flank skin upon restimulation with L. major infected BMDCs, is shown for immune mice treated with 500μg RB6-8C5 or 1A8. (TIF) [file ppat.1006349.s004.tif]

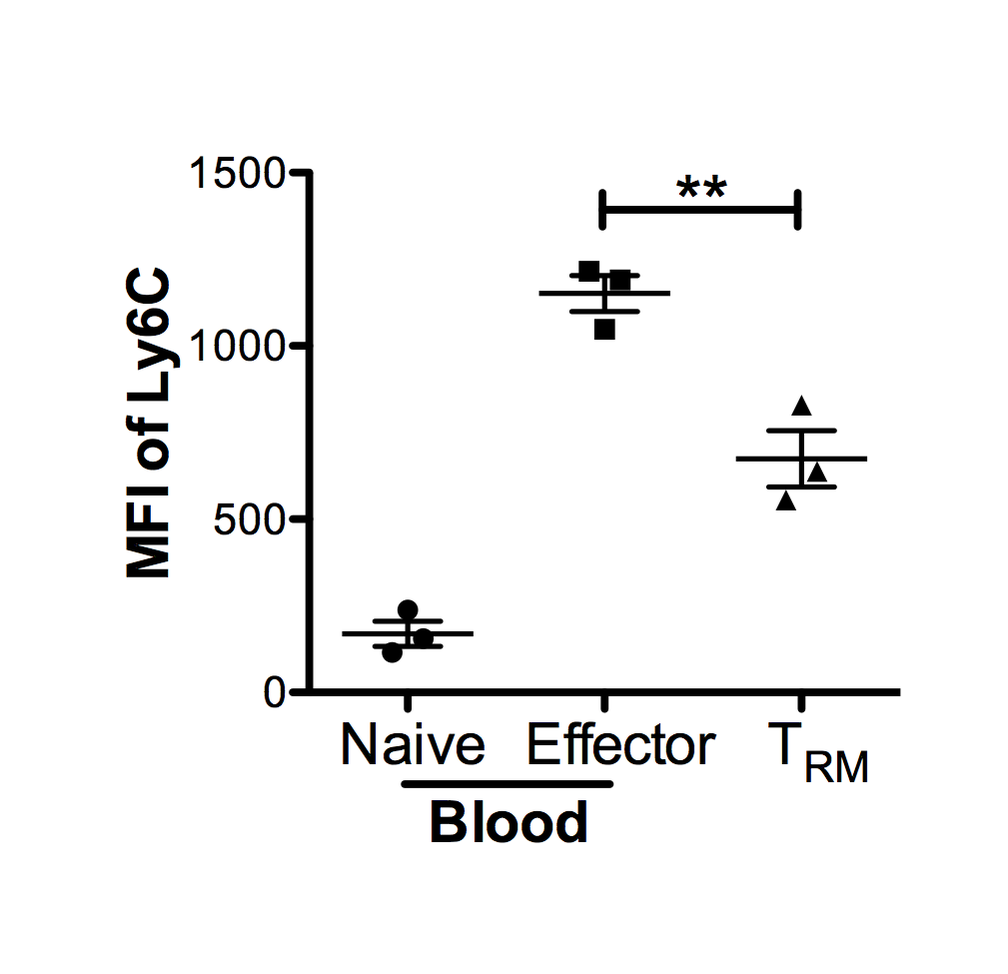

Supplement: S5 Fig — Comparison of Ly6C MFI on naïve or Ly6C+ effector cells from the blood and TRM cells from the flank, as represented by cells that produced IFNγ in response to restimulation with L. major infected BMDCs. (TIF) [file ppat.1006349.s005.tif]

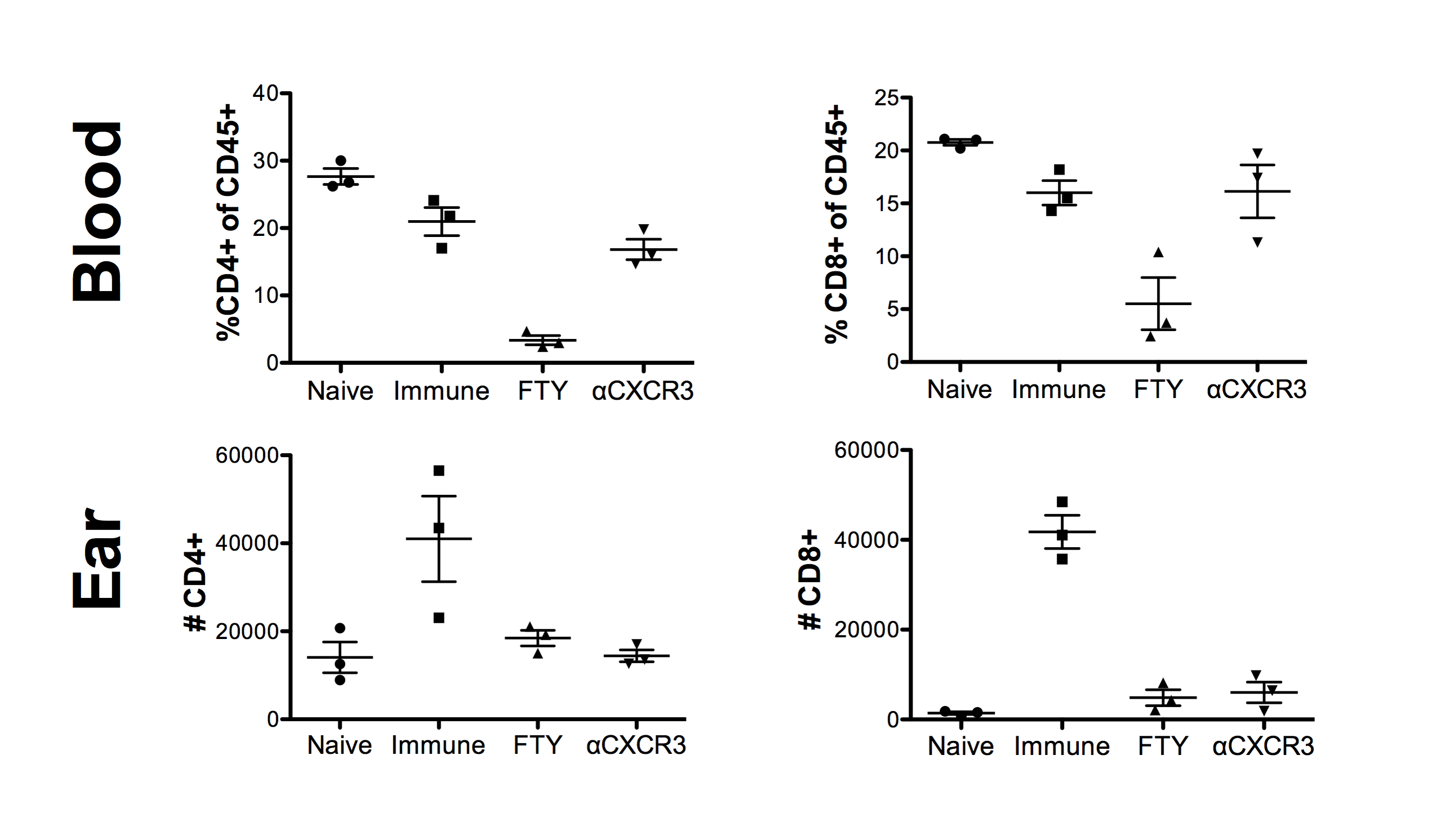

Supplement: S6 Fig — Frequency or number of CD4+ and CD8+ cells in the blood and challenged ear 72 hours after infection of FTY-720 or αCXCR3 treated immune mice are shown. (TIF) [file ppat.1006349.s006.tif]

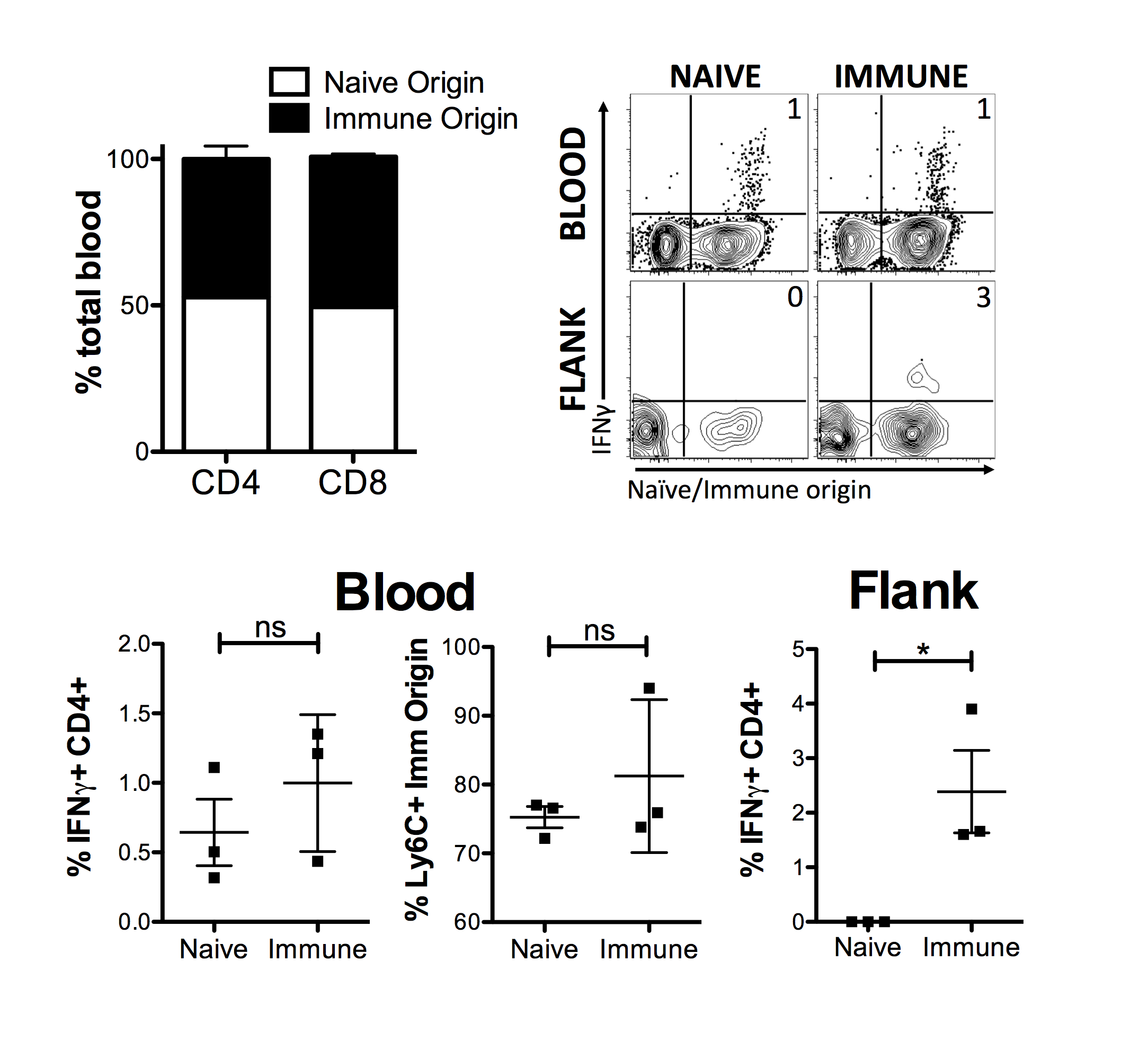

Supplement: S7 Fig — (Top left) Proportions of CD4+ and CD8+ T cells of naïve (white) or immune (black) origin found in naïve parabionts 2.5 weeks after joining. (Top right) Representative plots showing frequency of leishmania-specific, IFNγ+ cells in the blood and flank of naive and immune parabionts 2.5 weeks after surgery upon restimulation with L. major infected BMDCs. (Bottom) Combined data showing frequency of IFNγ+ cells in the blood and flank of naive and immune parabionts 2.5 weeks after surgery upon restimulation with L. major infected BMDCs, as well as frequency of immune origin Ly6C+ CD4+ T cells in naïve and immune parabionts. (TIF) [file ppat.1006349.s007.tif]
